# *N*-(2-Methyl­phenyl­sulfon­yl)propanamide

**DOI:** 10.1107/S1600536811014164

**Published:** 2011-04-22

**Authors:** K. Shakuntala, Sabine Foro, B. Thimme Gowda

**Affiliations:** a Department of Chemistry, Mangalore University, Mangalagangotri 574 199, Mangalore, India; b Institute of Materials Science, Darmstadt University of Technology, Petersenstrasse 23, D-64287 Darmstadt, Germany

## Abstract

In the title compound, C_10_H_13_NO_3_S, the conformations of the N—H and C=O bonds of the SO_2_—NH—CO—C segment are *anti* to each other, while the amide H atom is *syn* with respect to the *ortho*-methyl group in the benzene ring. The C—S—N—C torsion angle is −66.7 (2)°. The crystal structure features inversion-related dimers linked by pairs of N—H⋯O(S) hydrogen bonds.

## Related literature

For hydrogen-bonding modes of sulfonamides, see: Adsmond & Grant (2001 ▶). For our study of the effect of substituents on the structures of *N*-(ar­yl)-amides, see: Gowda *et al.* (2004 ▶); on the structures of *N*-(substitutedphenyl­sulfon­yl)-substitutedamides, see: Shakuntala *et al.* (2011**a* ▶,b* ▶) and on the oxidative strengths of *N*-chloro, *N*-aryl­sulfonamides, see: Gowda & Kumar (2003 ▶).
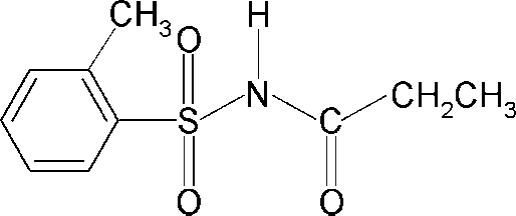


## Experimental

### 

#### Crystal data

C_10_H_13_NO_3_S
*M* *_r_* = 227.27
Monoclinic, 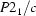

*a* = 8.3050 (8) Å
*b* = 13.339 (1) Å
*c* = 9.9948 (9) Å
β = 97.876 (9)°
*V* = 1096.78 (17) Å^3^
*Z* = 4
Mo *K*α radiation
μ = 0.28 mm^−1^
*T* = 293 K
0.44 × 0.32 × 0.16 mm

#### Data collection

Oxford Diffraction Xcalibur diffractometer with a Sapphire CCD detector
Absorption correction: multi-scan (*CrysAlis RED*; Oxford Diffraction, 2009 ▶) *T*_min_ = 0.886, *T*_max_ = 0.956
4277 measured reflections
2241 independent reflections
1607 reflections with *I* > 2σ(*I*)
*R*_int_ = 0.017

#### Refinement

*R*[*F* ^2^ > 2σ(*F* ^2^)] = 0.044
*wR*(*F* ^2^) = 0.108
*S* = 1.04
2241 reflections
140 parameters
1 restraint
H atoms treated by a mixture of independent and constrained refinement
Δρ_max_ = 0.20 e Å^−3^
Δρ_min_ = −0.35 e Å^−3^

### 

Data collection: *CrysAlis CCD* (Oxford Diffraction, 2009 ▶); cell refinement: *CrysAlis RED* (Oxford Diffraction, 2009 ▶); data reduction: *CrysAlis RED*; program(s) used to solve structure: *SHELXS97* (Sheldrick, 2008 ▶); program(s) used to refine structure: *SHELXL97* (Sheldrick, 2008 ▶); molecular graphics: *PLATON* (Spek, 2009 ▶); software used to prepare material for publication: *SHELXL97*.

## Supplementary Material

Crystal structure: contains datablocks I, global. DOI: 10.1107/S1600536811014164/ds2108sup1.cif

Structure factors: contains datablocks I. DOI: 10.1107/S1600536811014164/ds2108Isup2.hkl

Additional supplementary materials:  crystallographic information; 3D view; checkCIF report

## Figures and Tables

**Table 1:** Hydrogen-bond geometry (Å, °)

*D*—H⋯*A*	*D*—H	H⋯*A*	*D*⋯*A*	*D*—H⋯*A*
N1—H1*N*⋯O1^i^	0.82 (2)	2.08 (2)	2.901 (2)	173 (3)

Symmetry code: (i) 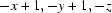.

**Fig. 1.**
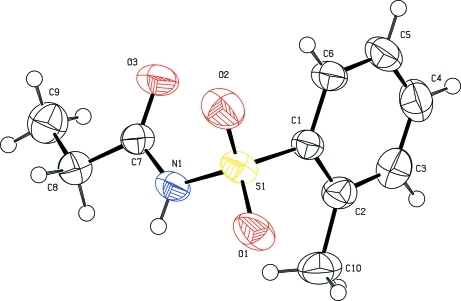
Molecular structure of the title compound, showing the atom- labelling scheme. Displacement ellipsoids are drawn at the 50% probability level.

**Fig. 2.**
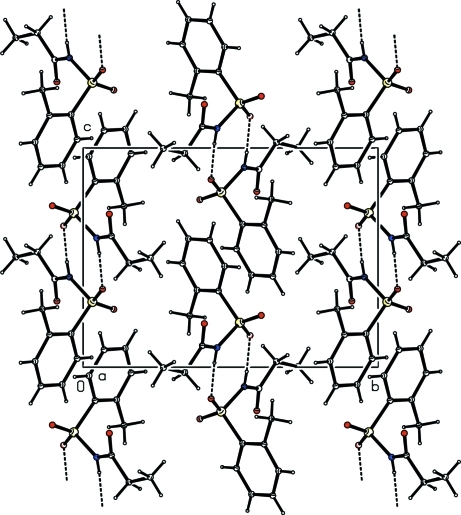
Molecular packing in the title compound. Hydrogen bonds are shown as dashed lines.

## supplementary crystallographic information

### Comment

The hydrogen bonding preferences of sulfonamides has been investigated (Adsmond & Grant, 2001). The nature and position of substituents play a significant role on the crystal structures and other aspects of *N*-(aryl)-amides and *N*-(aryl)-sulfonamides (Gowda *et al.*, 2003, 2004; Shakuntala *et al.*, 2011**a*,b*). As a part of studying the effects of substituents on the structures of this class of compounds, the structure of *N*-(2-methylphenylsulfonyl)-2-methylacetamide (I) has been determined (Fig. 1). The conformations of the N—H and C=O bonds of this segment in the structure are anti to each other, similar to that observed in *N*-(2-methylphenylsulfonyl)-acetamide (II) (Shakuntala *et al.*, 2011*b*) and *N*-(2-methylphenylsulfonyl)-2,2,2-trimethylacetamide (III) (Shakuntala *et al.*, 2011*a*). Further, the conformation of the amide H atom is *syn* to the *ortho*-methyl group in the benzene ring, similar to that observed between the amide H atom and the *ortho*-methyl group in (II) and (III).

The molecules in (I) are bent at the *S*-atom with a C—S—N—C torsion angle of -66.7 (2)°, compared to the values of -58.2 (2)° in (II) and -65.4 (2)° in (III).

In the crystal structure, the pairs of intermolecular N–H···O hydrogen bonds (Table 1) link the molecules through inversion-related dimers into chains running in the direction of *b*-axis. Part of the crystal structure is shown in Fig. 2.

### Experimental

The title compound was prepared by refluxing 2-methylbenzenesulfonamide (0.10 mole) with an excess of propanoyl chloride (0.20 mole) for one hour on a water bath. The reaction mixture was cooled and poured into ice cold water. The resulting solid was separated, washed thoroughly with water and dissolved in warm dilute sodium hydrogen carbonate solution. The title compound was reprecipitated by acidifying the filtered solution with glacial acetic acid. It was filtered, dried and recrystallized from ethanol. The purity of the compound was checked by determining its melting point. It was further characterized by recording its infrared spectra.

Prism like colourless single crystals of the title compound used in X-ray diffraction studies were obtained from a slow evaporation of an ethanolic solution of the compound.

### Refinement

The H atom of the NH group was located in a difference map and later restrained to the distance N—H = 0.86 (2) Å. The other H atoms were positioned with idealized geometry using a riding model with the aromatic C—H distance = 0.93 Å, methyl C—H = 0.96 Å, methylene C—H = 0.97 Å. All H atoms were refined with isotropic displacement parameters (set to 1.2 times of the *U*_eq_ of the parent atom).

### Crystal data

**Table:**

C_10_H_13_NO_3_S	*F*(000) = 480
*M**_r_* = 227.27	*D*_x_ = 1.376 Mg m^−^^3^
Monoclinic, *P*2_1_/*c*	Mo *K*α radiation, λ = 0.71073 Å
Hall symbol: -P 2ybc	Cell parameters from 1271 reflections
*a* = 8.3050 (8) Å	θ = 2.6–27.8°
*b* = 13.339 (1) Å	µ = 0.28 mm^−^^1^
*c* = 9.9948 (9) Å	*T* = 293 K
β = 97.876 (9)°	Prism, colourless
*V* = 1096.78 (17) Å^3^	0.44 × 0.32 × 0.16 mm
*Z* = 4	

### Data collection

**Table:**

Oxford Diffraction Xcalibur diffractometer with a Sapphire CCD detector	2241 independent reflections
Radiation source: fine-focus sealed tube	1607 reflections with *I* > 2σ(*I*)
graphite	*R*_int_ = 0.017
Rotation method data acquisition using ω and φ scans	θ_max_ = 26.4°, θ_min_ = 2.6°
Absorption correction: multi-scan (*CrysAlis RED*; Oxford Diffraction, 2009)	*h* = −10→10
*T*_min_ = 0.886, *T*_max_ = 0.956	*k* = −11→16
4277 measured reflections	*l* = −12→7

### Refinement

**Table:**

Refinement on *F*^2^	Primary atom site location: structure-invariant direct methods
Least-squares matrix: full	Secondary atom site location: difference Fourier map
*R*[*F*^2^ > 2σ(*F*^2^)] = 0.044	Hydrogen site location: inferred from neighbouring sites
*wR*(*F*^2^) = 0.108	H atoms treated by a mixture of independent and constrained refinement
*S* = 1.04	*w* = 1/[σ^2^(*F*_o_^2^) + (0.0396*P*)^2^ + 0.5309*P*] where *P* = (*F*_o_^2^ + 2*F*_c_^2^)/3
2241 reflections	(Δ/σ)_max_ < 0.001
140 parameters	Δρ_max_ = 0.20 e Å^−^^3^
1 restraint	Δρ_min_ = −0.35 e Å^−^^3^

### Special details

**Table:**

Experimental. CrysAlis RED (Oxford Diffraction, 2009) Empirical absorption correction using spherical harmonics, implemented in SCALE3 ABSPACK scaling algorithm.
Geometry. All e.s.d.'s (except the e.s.d. in the dihedral angle between two l.s. planes) are estimated using the full covariance matrix. The cell e.s.d.'s are taken into account individually in the estimation of e.s.d.'s in distances, angles and torsion angles; correlations between e.s.d.'s in cell parameters are only used when they are defined by crystal symmetry. An approximate (isotropic) treatment of cell e.s.d.'s is used for estimating e.s.d.'s involving l.s. planes.
Refinement. Refinement of *F*^2^ against ALL reflections. The weighted *R*-factor *wR* and goodness of fit *S* are based on *F*^2^, conventional *R*-factors *R* are based on *F*, with *F* set to zero for negative *F*^2^. The threshold expression of *F*^2^ > σ(*F*^2^) is used only for calculating *R*-factors(gt) *etc*. and is not relevant to the choice of reflections for refinement. *R*-factors based on *F*^2^ are statistically about twice as large as those based on *F*, and *R*- factors based on ALL data will be even larger.

### Fractional atomic coordinates and isotropic or equivalent isotropic displacement parameters (Å^2^)

**Table:**

	*x*	*y*	*z*	*U*_iso_*/*U*_eq_	
C1	0.6604 (2)	0.45905 (16)	0.3478 (2)	0.0403 (5)	
C2	0.5397 (3)	0.38655 (18)	0.3512 (2)	0.0456 (6)	
C3	0.5387 (3)	0.3371 (2)	0.4736 (3)	0.0586 (7)	
H3	0.4597	0.2886	0.4800	0.070*	
C4	0.6499 (3)	0.3571 (2)	0.5856 (3)	0.0622 (7)	
H4	0.6454	0.3221	0.6655	0.075*	
C5	0.7679 (3)	0.4288 (2)	0.5794 (2)	0.0584 (7)	
H5	0.8431	0.4427	0.6550	0.070*	
C6	0.7736 (3)	0.47990 (19)	0.4607 (2)	0.0484 (6)	
H6	0.8530	0.5284	0.4557	0.058*	
C7	0.8782 (3)	0.40395 (18)	0.0980 (2)	0.0461 (6)	
C8	0.9020 (3)	0.34442 (19)	−0.0249 (2)	0.0501 (6)	
H8A	0.9198	0.3903	−0.0967	0.060*	
H8B	0.8032	0.3072	−0.0548	0.060*	
C9	1.0421 (3)	0.2722 (2)	−0.0023 (3)	0.0646 (7)	
H9A	1.0251	0.2259	0.0679	0.078*	
H9B	1.1412	0.3086	0.0237	0.078*	
H9C	1.0495	0.2359	−0.0843	0.078*	
C10	0.4116 (3)	0.3615 (2)	0.2348 (3)	0.0633 (7)	
H10A	0.4601	0.3585	0.1530	0.076*	
H10B	0.3288	0.4122	0.2264	0.076*	
H10C	0.3641	0.2977	0.2506	0.076*	
N1	0.7295 (2)	0.45380 (16)	0.08882 (19)	0.0497 (5)	
H1N	0.664 (3)	0.4451 (19)	0.020 (2)	0.060*	
O1	0.5187 (2)	0.56595 (14)	0.14397 (17)	0.0616 (5)	
O2	0.8011 (2)	0.60402 (13)	0.23614 (19)	0.0673 (5)	
O3	0.9760 (2)	0.41109 (16)	0.19851 (18)	0.0681 (5)	
S1	0.67655 (7)	0.53152 (5)	0.20265 (6)	0.04798 (19)	

### Atomic displacement parameters (Å^2^)

**Table:**

	*U*^11^	*U*^22^	*U*^33^	*U*^12^	*U*^13^	*U*^23^
C1	0.0367 (11)	0.0465 (13)	0.0363 (11)	0.0061 (10)	0.0005 (9)	−0.0056 (10)
C2	0.0397 (12)	0.0487 (14)	0.0474 (13)	0.0064 (10)	0.0020 (10)	−0.0065 (11)
C3	0.0538 (14)	0.0620 (16)	0.0623 (17)	0.0026 (13)	0.0164 (12)	0.0013 (14)
C4	0.0675 (17)	0.0768 (19)	0.0443 (14)	0.0150 (15)	0.0145 (13)	0.0073 (14)
C5	0.0563 (15)	0.0782 (19)	0.0376 (13)	0.0161 (14)	−0.0048 (11)	−0.0056 (13)
C6	0.0405 (12)	0.0595 (15)	0.0426 (12)	0.0029 (11)	−0.0034 (9)	−0.0057 (11)
C7	0.0381 (11)	0.0577 (15)	0.0414 (12)	−0.0047 (11)	0.0019 (10)	0.0041 (11)
C8	0.0447 (13)	0.0606 (16)	0.0448 (13)	−0.0051 (11)	0.0055 (10)	0.0008 (12)
C9	0.0612 (16)	0.0646 (17)	0.0684 (18)	0.0062 (14)	0.0103 (14)	−0.0021 (15)
C10	0.0477 (14)	0.0692 (17)	0.0695 (17)	−0.0068 (13)	−0.0049 (12)	−0.0150 (14)
N1	0.0429 (11)	0.0684 (14)	0.0352 (10)	0.0068 (10)	−0.0046 (8)	−0.0063 (10)
O1	0.0666 (11)	0.0681 (11)	0.0458 (10)	0.0258 (9)	−0.0083 (8)	−0.0008 (8)
O2	0.0823 (13)	0.0584 (11)	0.0592 (11)	−0.0211 (10)	0.0033 (9)	−0.0005 (9)
O3	0.0474 (10)	0.0995 (15)	0.0524 (11)	0.0089 (10)	−0.0105 (8)	−0.0127 (10)
S1	0.0515 (3)	0.0502 (4)	0.0396 (3)	0.0034 (3)	−0.0036 (2)	0.0002 (3)

### Geometric parameters (Å, °)

**Table:**

C1—C6	1.394 (3)	C7—C8	1.499 (3)
C1—C2	1.397 (3)	C8—C9	1.504 (3)
C1—S1	1.763 (2)	C8—H8A	0.9700
C2—C3	1.391 (3)	C8—H8B	0.9700
C2—C10	1.503 (3)	C9—H9A	0.9600
C3—C4	1.377 (4)	C9—H9B	0.9600
C3—H3	0.9300	C9—H9C	0.9600
C4—C5	1.377 (4)	C10—H10A	0.9600
C4—H4	0.9300	C10—H10B	0.9600
C5—C6	1.375 (3)	C10—H10C	0.9600
C5—H5	0.9300	N1—S1	1.643 (2)
C6—H6	0.9300	N1—H1N	0.822 (16)
C7—O3	1.206 (3)	O1—S1	1.4363 (17)
C7—N1	1.395 (3)	O2—S1	1.4219 (18)
			
C6—C1—C2	121.7 (2)	C7—C8—H8B	108.8
C6—C1—S1	115.96 (18)	C9—C8—H8B	108.8
C2—C1—S1	122.38 (16)	H8A—C8—H8B	107.7
C3—C2—C1	116.2 (2)	C8—C9—H9A	109.5
C3—C2—C10	119.1 (2)	C8—C9—H9B	109.5
C1—C2—C10	124.7 (2)	H9A—C9—H9B	109.5
C4—C3—C2	122.6 (3)	C8—C9—H9C	109.5
C4—C3—H3	118.7	H9A—C9—H9C	109.5
C2—C3—H3	118.7	H9B—C9—H9C	109.5
C3—C4—C5	120.0 (3)	C2—C10—H10A	109.5
C3—C4—H4	120.0	C2—C10—H10B	109.5
C5—C4—H4	120.0	H10A—C10—H10B	109.5
C6—C5—C4	119.4 (2)	C2—C10—H10C	109.5
C6—C5—H5	120.3	H10A—C10—H10C	109.5
C4—C5—H5	120.3	H10B—C10—H10C	109.5
C5—C6—C1	120.1 (2)	C7—N1—S1	125.02 (16)
C5—C6—H6	120.0	C7—N1—H1N	117.6 (18)
C1—C6—H6	120.0	S1—N1—H1N	117.3 (18)
O3—C7—N1	120.4 (2)	O2—S1—O1	118.19 (12)
O3—C7—C8	125.1 (2)	O2—S1—N1	109.70 (11)
N1—C7—C8	114.42 (19)	O1—S1—N1	103.59 (10)
C7—C8—C9	113.7 (2)	O2—S1—C1	108.31 (11)
C7—C8—H8A	108.8	O1—S1—C1	110.06 (10)
C9—C8—H8A	108.8	N1—S1—C1	106.35 (11)
			
C6—C1—C2—C3	0.3 (3)	N1—C7—C8—C9	−166.1 (2)
S1—C1—C2—C3	−178.22 (17)	O3—C7—N1—S1	4.7 (4)
C6—C1—C2—C10	178.7 (2)	C8—C7—N1—S1	−174.80 (17)
S1—C1—C2—C10	0.1 (3)	C7—N1—S1—O2	50.2 (2)
C1—C2—C3—C4	−0.4 (4)	C7—N1—S1—O1	177.3 (2)
C10—C2—C3—C4	−178.8 (2)	C7—N1—S1—C1	−66.7 (2)
C2—C3—C4—C5	0.3 (4)	C6—C1—S1—O2	−4.0 (2)
C3—C4—C5—C6	−0.1 (4)	C2—C1—S1—O2	174.62 (18)
C4—C5—C6—C1	0.1 (4)	C6—C1—S1—O1	−134.60 (17)
C2—C1—C6—C5	−0.2 (3)	C2—C1—S1—O1	44.0 (2)
S1—C1—C6—C5	178.44 (18)	C6—C1—S1—N1	113.82 (18)
O3—C7—C8—C9	14.4 (4)	C2—C1—S1—N1	−67.5 (2)

### Hydrogen-bond geometry (Å, °)

**Table:**

*D*—H···*A*	*D*—H	H···*A*	*D*···*A*	*D*—H···*A*
N1—H1N···O1^i^	0.82 (2)	2.08 (2)	2.901 (2)	173 (3)

Symmetry codes: (i) −*x*+1, −*y*+1, −*z*.
